# Arcobacter Butzleri in an AIDS Patient

**DOI:** 10.1155/2022/6983094

**Published:** 2022-07-06

**Authors:** Trevor Hwee Yong Tan, Sai Meng Tham, Paul Anatharajah Tambyah

**Affiliations:** ^1^National University Hospital, Department of Internal Medicine, Division of Medicine, Singapore; ^2^National University Hospital, Department of Infectious Diseases, Division of Medicine, Singapore

## Abstract

**Background:**

*Arcobacter butzleri (A. butzleri)* is an emerging enteric pathogen increasingly identified in Europe and is likely under-reported in other global regions. We describe to our knowledge the first case report of *A. butzleri* in an AIDS patient, along with the first documented local (Singapore) case of *A. butzleri* infection. *Case Presentation*. A 38-year-old AIDS patient presented with diarrhoea of 2 weeks' duration. Stool cultures yielded *A. butzleri*. The patient was treated with 3 days of ciprofloxacin with clinical resolution of diarrhoea.

**Conclusion:**

*A. butzleri* is likely to be present, although under-reported in AIDS patients, and it should be noted as a pathogen of increasing significance.

## 1. Introduction

Patients with human immunodeficiency virus (HIV) are more prone to acquiring enteric infections from food-borne and zoonotic pathogens [1]. *Arcobacter butzleri (A. butzleri)* is a *Campylobacter*-like organism of the genus *Arcobacter* [2] that has been increasingly identified as an emerging enteric pathogen [2, 3].

We report a case of *A. butzleri* infection in an HIV-positive patient with acquired immunodeficiency syndrome (AIDS). This is likely to be the first case reported of its kind in an AIDS patient and in Singapore based on literature review. Clinicians need to be aware of this pathogen and its treatment and complications in immunocompromised hosts.

## 2. Case Presentation

A 38-year-old man with a history of HIV infection diagnosed in 2016 but only on intermittent antiviral treatment presented with symptoms of acute diarrhoea lasting 2 weeks. The diarrhoeal episodes occurred about ten to twenty times per day and stools were neither bloody nor mucoid. The diarrhoeal episode was associated with clinically significant loss of weight and cramping abdominal pain. The patient had been on boosted protease inhibitor (darunavir/ritonavir) monotherapy for 6 months prior to admission. There was no recent travel history or intake of raw or uncooked food, and he had worked as a freelance architect prior to losing his job at the start of the pandemic. Clinical examination revealed a hypovolemic fluid state with significant cachexia, while bowel sounds were hyperactive. There was no abdominal tenderness, and digital rectal examination was unremarkable.

**Figure 1:**
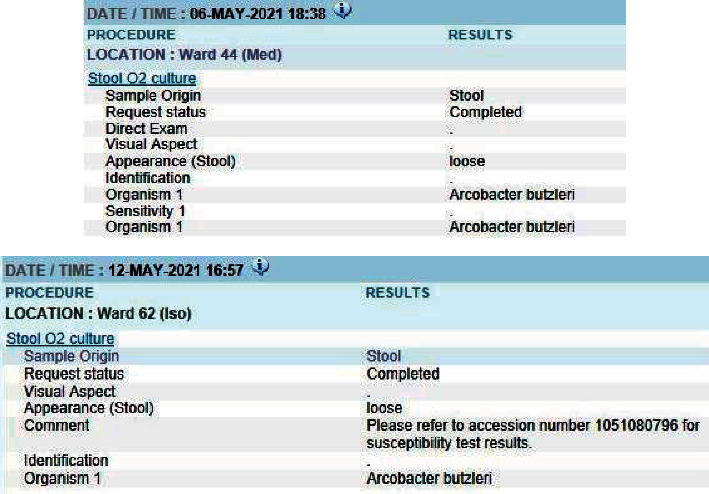
Stool culture reports indicating presence of *A. butzleri.*

**Figure 2:**
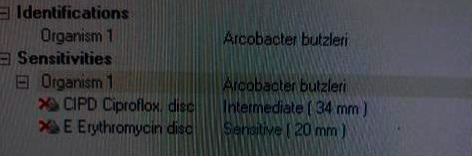
Sensitivities of identified *A. butzleri* in our patient.

Laboratory investigations showed evidence of mild leukocytosis (10.03 × 10^9^/L) with neutrophilia (9.36 × 10^9^/L); his absolute CD4 count was <20. His viral load was 87 × 10^6^ U/mL. There was no elevated creatinine to suggest acute kidney injury, although his urea level was slightly elevated, indicating dehydration. Blood cultures were negative for bacterial organisms. Stool cultures were positive for *A. butzleri* on 2 separate occasions (Figure 1). Sensitivity testing yielded intermediate sensitivities to ciprofloxacin (34 mm) and high sensitivities to erythromycin (20 mm) (Figure 2).

**Figure 3:**
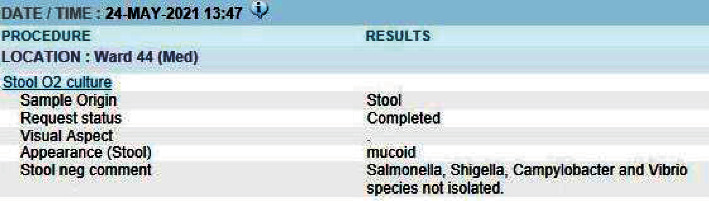
Stool culture report after 3-day course of ciprofloxacin.

The patient was subsequently treated presumptively with 3 days of oral ciprofloxacin 500 mg twice daily with complete clinical resolution of diarrhoea and abdominal discomfort. Repeat stool cultures after the completion of antibiotics were also negative (Figure 3). Unfortunately, he developed other infective complications such as nosocomial pneumonia, cytomegalovirus (CMV) colitis, and disseminated *Mycobacterium avium* complex (MAC) infection. He passed away from respiratory failure from severe pneumonia 4 weeks after admission.

## 3. Discussion and Conclusions

*A. butzleri* is a Gram-negative, motile, aerotolerant microbe of the *Epislonproteo bacteria* class and *Campylobacteraceae* family [2] and is one of 27 recognised species of *Arcobacter* characterised to date [3]. It was first isolated in aborted bovine foetuses [4] and was later linked to causing reproductive disorders and late-term abortions in cattle, pigs, and sheep [5]. It has been present in a range of commonly consumed meat including poultry and red meat [6, 7] and contains virulence genes such as *ciaB* (*Campylobacter* invasive antigen B) and *hecB* (encoding a haemolysin activation protein) [8], in addition to possessing adhesive and invasive properties toward multiple human cell lines [9]. These virulence genes are nonetheless not routinely tested in our laboratory and thus were not tested in this patient's specimen.

As a result, *A. butzleri* has been identified as an emerging enteropathogen and a zoonotic agent of increasing significance [10]. Taylor et al. identified *A. butzleri* in 2.4% of diarrhoeal samples in Thai children in 1991 [11], while several sporadic outbreaks of *A. butzleri* gastroenteritis have been observed in Europe and South Africa across the 1990s and 2000s [12–14]. Gastrointestinal infection and colonisation with *A. butzleri* have since demonstrated increasing prevalence; *A. butzleri* was identified as a causative organism for 24 out of 4636 cases of gastroenteritis in a prospective study in Germany [15], while several studies have identified *A. butzleri* to be amongst the most frequently isolated *Campylobacteraceae* strain in human clinical samples [16, 17].

While gastrointestinal infections with *A. butzleri* are becoming more common, severe infections such as bacteraemia remain rare. A case of *A. butzleri* bacteraemia was observed in a neonate in the United Kingdom [18], while in Hong Kong, two cases of *A. butzleri* bacteraemia have been reported, both patients with underlying diseases of liver cirrhosis and gangrenous appendicitis, respectively [19, 20]. A single case of *Arcobacter* causing peritonitis has been reported in a patient on peritoneal dialysis, occurring after repositioning of a Tenckhoff catheter [21]. Case studies documenting *A. butzleri* infection, ranging from gastrointestinal infections to bacteraemia, are apprised in Table 1. Despite the well-documented phenomenon of enteral infections with *A. butzleri*, there have yet to be documented cases of infection in HIV patients, although it is highly likely that the true global prevalence is underestimated. We believe that this is the first documented case of its kind, in addition to being the first in Singapore to document a case of *A. butzleri* infection locally.

Differential diagnoses to be considered for this patient would include cytomegalovirus (CMV) colitis, *Mycobacterium avium* enteric infection, as well as HIV-associated enteropathy [24], along with other *Campylobacter* species that are found more commonly in HIV patients [25, 26].

The majority of *A. butzleri* infections are self-limiting and do not require treatment with antibiotics unless clinically indicated, such as in cases of severe and persistent symptoms [17], as in our patient. Despite its status as an emerging pathogen, data on antibiotic susceptibility of *A. butzleri* remain sparse. The most optimal antibiotic choice for treatment of *A. butzleri* is still unclear. The species has been classified as a potential multidrug resistant (MDR) organism as it has demonstrated varying levels of susceptibility toward different classes of agents [27]. In a Belgian antibiotic susceptibility study of 63 strains of *A. butzleri*, most strains (87%) were susceptible to ciprofloxacin (MIC_90_ 32 mg/L), while moderate levels (36%) of resistance to doxycycline (MIC_90_ 4 mg/L) were reported [22]. Combined resistance to erythromycin and doxycycline was observed in 10 out of 13 strains of *A. butzleri* [22]. Bruckner et al. also showed that ciprofloxacin was the most effective antibiotic agent for treatment of *A. butzleri* as compared to other antibiotics such as azithromycin [15], which are commonly used for *Campylobacter*. However, another meta-regression analysis revealed emerging resistance of Arcobacter species against fluoroquinolones [28]; this was corroborated in the case of our patient, whose specimens instead demonstrated the greater efficacy of erythromycin as opposed to ciprofloxacin (Figure 2).

In summary, *A. butzleri* is an emerging pathogen which is recognised increasingly in Europe and probably underrecognised in the rest of the world. Ours is the first documented case of *A. butzleri* in Singapore and possibly the first in an HIV patient. Clinicians should recognise the pathogenicity of *A. butzleri* in immunocompromised hosts and should always send stool cultures to test for bacterial strains especially in cases of persistent diarrhoea. Given that *A. butzleri's* antibiotic susceptibility pattern is different from *Campylobacter's,* laboratories in centres treating individuals with HIV should also be alert to this treatable pathogen to reduce morbidity.

## Figures and Tables

**Table 1:** Case series of documented *A. butzleri* infections.

Case studies	Treatment	Reference
Diarrhoea in rural children, Thailand (93)	Not stated	[11]
Gastrointestinal symptoms campylobacter-like infections, France (29)	2 treated with oral (PO) fluoroquinolones (ciprofloxacin), 1 treated with PO co-amoxiclav	[17]
Enteritis (46), of which 19 had acute gastroenteritis, 30 had co-existing conditions and 8 had chronic colitis, Belgium	Not stated	[22]
Gastroenteritis (24) out of 3884 outpatients and 752 inpatients	Not stated; recommendations were for fluoroquinolones	[15]
Bactaraemia in an immunocompromised host (1) (85 y/o CLL on idelalisib)	Intravenous (IV) piperacillin-tazobactam and vancomycin	[23]
Bactaraemia in a neonate (1) causing neonatal sepsis	Not stated	[18]
Bactaraemia in a patient with acute gangrenous appendicitis (1)	IV cefuroxime and metronidazole	[20]
Bactaraemia in a liver cirrhosis patient (1)	IV cefuroxime	[19]
Peritoneal dialysis (PD) peritonitis (1)	IV ticarcillin-clavulanate x 2 weeks	[21]

## Data Availability

Data sharing is not applicable to this article as no datasets were generated or analysed during the current study.

## Abbreviations

*A. butzleri*: Arcobacter butzleri.
